# Diet quality and its relationship with iodine status in pregnant women living in a Brazilian region where table salt is iodine-fortified according to public health policies

**DOI:** 10.20945/2359-4292-2024-0164

**Published:** 2025-06-27

**Authors:** Annie Schtscherbyna, Débora Ayres Saraiva, Nathalie Silva de Morais, Carolina Martins Corcino, Tatiana Martins Benvenuto Louro Berbara, Mario Vaisman, Paula Martins Horta, Patrícia de Fátima dos Santos Teixeira

**Affiliations:** 1 Programa de Pós-graduação em Endocrinologia, Faculdade de Medicina, Universidade Federal do Rio de Janeiro, Rio de Janeiro, RJ, Brasil; 2 Departamento de Nutrição, Universidade Federal de Minas Gerais, Belo Horizonte, MG, Brasil

**Keywords:** Brazil, diet quality, healthy eating index, iodine status, pregnancy

## Abstract

**Objective:**

Conditions related to iodine status (IS) during pregnancy should be addressed to improve public health strategies. The aim of this study was to analyze the relationship between diet quality (DQ), assessed by the adapted Healthy Eating Index-2015 (HEI-2015), and IS in pregnant women from a Brazilian coastal state where public health policies assure iodine-fortified table salt in concentrations ranging from 15 to 45 mg/kg.

**Subjects and methods:**

In a cross-sectional study, 199 participants were evaluated on three different days during their first trimester of pregnancy. At every visit, a urinary spot sample was requested to assess the urinary iodine concentration (UIC), and a 24-hour dietary recall related to the time at which the urine was collected was also performed. Total and component scores were estimated for the HEI-2015. The association between DQ and the IS was evaluated, considering an adequate UIC (150-249 µg/L) as the reference group (RG).

**Results:**

The median total score for the HEI-2015 was 45.8 points, which was slightly better (48.1 points) in the insufficient UIC (UIC <150-249 µg/L) than in the RG (41.3 points). In the “more than adequate” and “excessive” IS groups, the scores were 40.7 and 44.6 points, respectively.

**Conclusion:**

Pregnant women with insufficient IS had better DQ and higher consumption of fruits and vegetables, as did those with lower intake of refined grains. We suppose that these groups benefit from iodine supplementation during pregnancy even when they live in a coastal state where table salt is fortified with iodine. In contrast, excessive UIC was related to worse intake of “whole fruits”, “total fruits” and “total vegetables”. The results suggest that diet quality is related to iodine status in the studied population. Pregnant women with better diet quality, especially those with high consumption of total protein foods, fruits and vegetables and low consumption of refined grains, may be at risk for iodine insufficiency. The necessity of iodine supplementation for pregnant women should be better explored even in regions where iodine-fortified table salt is adopted as a public health policy.

## INTRODUCTION

Public health policies that ensure iodine-fortified table salt for the population contribute to reducing the risk of iodine deficiency and its consequences, such as endemic goiter, intellectual disability, hypo- and hyperthyroidism, and thyroid nodules, among other unfavorable outcomes (^1^,^2^). Furthermore, assessing its efficacy, especially in critical populations, such as pregnant women, is crucial (^1^,^2^). It is important to monitor the iodine content in table salt and the median urinary iodine concentration (UIC) of specific populations, especially children and pregnant women, to ensure the efficacy of the program (^1^,^2^). The median UIC of a population is considered a good indicator of iodine status and reflects the iodine available for metabolism since this micronutrient is completely eliminated by renal clearance. Recently, it was found that the median UIC of pregnant women from a coastal Brazilian state exposed to a reduction in the concentration of iodine in table salt, according to local governmental requirements, was adequate and in accordance with the World Health Organization (WHO) recommendations (^3^). Additionally, most table salt samples collected contained the required iodine concentration of 15-45 mg/kg (^3^). Nevertheless, high variability in the UIC of pregnant women was detected, with 25.3% of all 629 collected urinary samples showing values < 150 µg/L, which is compatible with insufficient iodine status (^3^). In contrast, 6.2% showed excessive iodine concentrations (>500 µg/L) (^3^). Given that both insufficient and excessive iodine lead to risks during pregnancy, it became necessary to explore factors that could explain this high variability. In attempting to promote adequate public health policies, it would be important to define which women would require iodine supplementation during pregnancy and to detect those at high risk for supplementation. Risk factors for iodine insufficiency and excess in this population are related to age, parity, and body mass index (BMI) (^3^). Because BMI has been positively associated with UIC, it was speculated that diet quality could be a factor related to iodine status (^3^).

The present study intended to evaluate how adding the assessment of diet quality to prenatal evaluation might be an additional instrument to estimate the risk for iodine insufficiency or excess in pregnant women. Since UIC evaluation is not a feasible routine laboratory method for prenatal evaluation, we speculate that the assessment of diet quality could be an alternative and indirect method to identify pregnant women at risk of insufficient UIC. In fact, UIC measurements to determine iodine status should not be applied outside epidemiologic studies.

In recent decades, an energy-dense nutrient-poor ‘Western’ dietary pattern, typically characterized by a low intake of fruits and vegetables combined with a high intake of ultra-processed foods (rich in fat and sugar), has become prevalent globally (^4^,^5^). This dietary pattern has been associated with the development of several diseases, such as high blood pressure, diabetes, and obesity, in the global population; this is especially concerning in some vulnerable subgroups, such as pregnant women and fetuses (^6^). The quality of food intake and nutrient input during this period are important for pregnant health and fetal development. One of the most important nutrients required during pregnancy is iodine (^7^). Furthermore, during pregnancy, women require greater amounts of iodine intake because of the increase in thyroid demand since several physiological changes lead to thyroid enlargement and hyperfunction (^7^).

In pregnant women, the impact of maternal iodine deficiency is related to maternal thyroid disorders, adverse pregnancy outcomes, impaired fetal nutritional status, fetal development (^8^), and children with a lower IQ. It has also been reported that not only deficiency, but also excess iodine may have a negative impact on pregnant women’s health and consequently on newborns. Associations between excessive iodine intake and thyroid autoimmunity and subclinical hypothyroidism have been reported in pregnant individuals (^9^,^10^). Furthermore, the present group previously reported an increased risk for gestational diabetes (GDM) and hypertensive disorders of pregnancy (HDP) when the UIC was ≥ 250 µg/L (^11^). In contrast, a shorter infant birth length was related to UICs < 150 µg/L (^11^).

The World Health Organization (WHO) recommends that iodine intake in pregnant women should be almost twice that of the general population, i.e., 250 µg/day *vs.* 100-150 µg/day (^12^). Therefore, this group needs special attention in relation to the nutritional status of this mineral (^12^).

A precise analysis of iodine ingestion is unfeasible because food iodine quantity depends on several elements, such as the soil quality where it was grown, the season when it was harvested, the food’s proximity to the sea, and the type of irrigation used (^13^). Additionally, people do not eat isolated nutrients but rather meals consisting of a variety of foods with complex nutrient combinations.

The evaluation of diet quality is a way of monitoring a population’s adherence to dietary guidelines. Recently, Souza and cols. (^4^) proposed an adaptation to the Healthy Eating Index-2015 (HEI-2015) for the Brazilian population. This proposal is in accordance with the Brazilian Dietary Guidelines that recommend avoiding ultraprocessed food products and prioritizing unprocessed and minimally processed foods (natural foods that have been somewhat altered before being purchased, including grains that are dried, polished, or ground as grits or flour; roots, tubers, and washed vegetables; refrigerated or frozen meat; and pasteurized milk) (^14^).

There are missing data regarding the association between diet quality and iodine status in pregnant women. Therefore, the aim of this study was to analyze the relationship between the adapted Healthy Eating Index-2015 (HEI-2015) and iodine status in pregnant women from a Brazilian coastal state where public health policies assure iodine-fortified table salt. We hypothesized that women with better diet quality may present with iodine insufficiency due to lower salt consumption.

## SUBJECTS AND METHODS

This was a cross-sectional evaluation of a population included in a prospective cohort. The research was approved by the local ethical committee (CAAE: 22546213.0.0000.5275), and all participants provided informed consent.

The participants were pregnant women aged ≥ 18 and ≤ 35 years in the first 12 weeks of gestation who attended prenatal appointments at one of four selected public basic health care units in urban areas of the municipality of Rio de Janeiro. The inclusion period was from May 2014 to February 2017. The participants had no previous history of thyroid or other chronic diseases. Patients with multifoetal pregnancies were excluded, as were those taking any kind of supplement containing iodine.

All pregnant women who fulfilled the inclusion criteria were invited to participate in the study during their first prenatal appointment. Three research visits were scheduled during one week of their first trimester, on nonconsecutive days, to assess three spot urine samples from each participant on separate days. For each urinary sample collected, a 24-hour dietary recall (24HR) was obtained. At the first visit, general clinical and pregnancy medical history assessments and physical examinations were performed.

The following information was obtained at enrollment: maternal age, gestational age, previous pregnancies and deliveries, current alcohol consumption, and current weight and height. BMI was calculated and classified according to gestational age (^15^).

Urinary iodine concentrations were determined via inductively coupled plasma-mass spectrometry. Iodine status was classified according to the WHO guidelines (^12^): severe insufficiency (<50 µg/L), mild-moderate insufficiency (50-149 µg/L), sufficiency (150-249 µg/L), more than adequate (250-499 µg/L), or excessive (≥500 µg/L). The samples were processed at Diagnosticos da America SA, a laboratory that is registered with the PALC program. Despite not being registered in the EQUIP program, an alternative control program evaluating the interlaboratory coefficient of variation was applied as previously reported (^11^).

As previously described, each urinary sample collected was accompanied by a 24HR. The participants were asked to recall what they had eaten in the past 24 hours, according to the time at which they collected the urine. These interviews were conducted by a nutritionist via the 5-stage multiple-pass interviewing technique (^16^). Portion sizes were estimated via common household measurements such as cups, glasses, bowls, teaspoons, and tablespoons.

The dietary data were entered into software that automatically converted the household measures into standard measures of weight and volume, such as grams and milliliters (^17^). To determine the nutritional value of each food and beverage recorded, a food composition database was developed based on compiled dietary data, mainly from the Brazilian Food Composition Tables (^18^) and the database of the Nutrition Data System for Research (^17^).

Diet quality was evaluated via the adapted HEI-2015 for the Brazilian population, in accordance with the Brazilian Dietary Guidelines (^4^). In this index, 13 components are analyzed: nine adequacy components – “total fruits”, “whole fruits”, “total vegetables”, “greens”, “whole grains”, “dairy”, “total protein foods”, “seafood and plant proteins”, and “fatty acids”) – and four moderation components – “refined grains”, “sodium”, “added sugars”, and “saturated fats”). All the components are density-based. With respect to adequacy components, higher scores are obtained with higher intake of such components, which represent a marker of better diet quality. In contrast, for moderation components, maximum scores (or better diet quality) are obtained with less intake of such components. The scores were then categorized, as previously suggested (^19^), into “high score” (90-100 points) and “low score” (0.0-49.9 points), considering, respectively, the better and worst scores for each component as well as the total HEI-2015 score (^19^). The aim was to compare the subgroup with a “high score” with those not fulfilling this criterion and thereafter to compare the group with a “low score” via the same approach. Those in an intermediary range (50-89.9 points) were not evaluated in a separate manner.

The mixed foods were broken into their component ingredients and then assigned to the appropriate adapted HEI-2015 category. Food and beverage quantities were converted into cups in accordance with the US Department of Agriculture Food Composition Databases (^20^).

SPSS version 21 was used for the statistical analysis. The Kolmogorov-Smirnov test indicated that no continuous variable had a normal distribution of data in the studied population. The adapted HEI-215 total score was compared among the iodine status categories. Additionally, the proportions of subjects who achieved the maximum and minimum scores for each diet quality component were calculated. Spearman’s correlation index was used to test which continuous variables were correlated with BMI and age.

Continuous variables are expressed as medians (minimum-maximum) and were compared between two groups via the Mann-Whitney test or between three or more groups via the Kruskal-Wallis test. The data were subsequently compared in a post hoc analysis via the Dunn test for multiple comparisons.

Categorical variables are described as frequencies and were explored among groups via the chi-square test. We also used the adjusted chi-square test for comparisons between subgroups, considering a p value < 0.0125 as significant in the four subgroups, with the aim of controlling for alpha error. P values above this value and < 0.10 were considered borderlines. Logistic binary regression was thereafter applied for multivariate analysis (Table 3), considering the reference group as those with adequate UIC. The variables included in the model were specific HEI category, age, parity, and BMI. The odds ratios (ORs) and 95% confidence intervals (95% CIs) were estimated. A p value < 0.05 was considered significant, and a p value of 0.05-0.10 was considered borderline in this context.

## RESULTS

A total of 418 UIC samples (with their respective 24HRs) from 199 women with a mean gestational age of 9.0 weeks were included in the analyses. There were 246 samples from 82 women who provided 3 samples, 110 from women who provided 2 samples, and 62 from women who provided only one sample.

**Figure 1:**
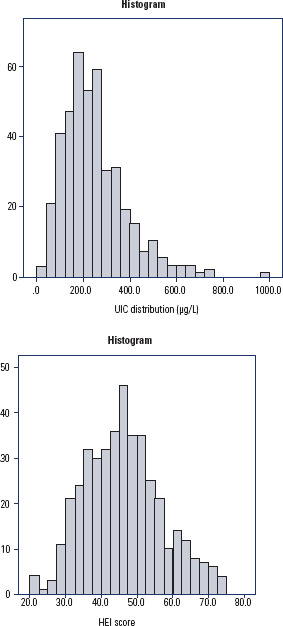
Histograms with the urinary iodine concentration and Healthy Eating Index-2015 scores distribution in the pregnant women (Rio de Janeiro, 2014-2017).

HEI: Healthy Eating Index-2015; UIC: urinary iodine concentration.

When all the urine samples collected (n = 418) were considered, the median UIC was 226.6 µg/L, and the histogram of the UIC distribution is shown in Figure 1. The frequency of samples in each subgroup, according to their iodine status (IS), was as follows: severe insufficiency, 1.5%; mild-moderate insufficiency, 22.7%; sufficiency, 34.9%; more than adequate, 35.2%; and excessive, 5.7%. To allow statistical analysis, the “severe insufficiency” and “mild-moderate insufficiency” groups were merged as “insufficient”.

The determination of clinical aspects, other than the HEI score, related to each IS was not the main objective of this study; however, the descriptions of the studied clinical characteristics, such as age, gestational age, BMI, and alcohol consumption status, according to the median UIC of the participants are shown in Supplementary Table 1.

On average, the women were aged 27.5 years and had a current BMI of 24.6 kg/m^2^. Almost half of the women were classified as having a normal weight (42.8%) and were nulliparous (47.9%). Almost half of the participants (48%) were in their first pregnancy, and only 4 participants were considered alcohol drinkers (1.0%).

**Figure 2:**
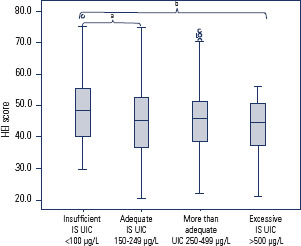
Boxplot’s graphic comparing Healthy Eating Index-2015 scores among the different subgroups, according to its iodine status in pregnant women (Rio de Janeiro, 2014-2017).

In terms of diet quality, the median total score for the adapted HEI-2015 in the studied group was 45.8 points (20.0-79.0), and the histogram of the HEI-2015 score distribution is shown in Figure 1. Compared with that in the reference group (adequate IS), it was slightly greater (48.1 points) in the iodine-insufficient group and in comparison with the excessive IS group (Figure 2). In the “more than adequate” and “excessive” iodine status subgroups, the scores were 40.7 and 44.6, respectively (Figure 2).

IS: iodine status; p=0.08 comparing all groups (Kruskal-Wallis test); a: p=0.02; b: p=0.04.

Considering the entire group, no woman had a high score (90-100 points) on the HEI-2015, as a whole, in any of the applied 24HRs. However, scores below 50.0 points, reflecting low scores or insufficient diet quality, were detected in 65.8% of the studied samples. A description of all the clinical characteristics and comparisons between the subgroups with or without low scores on the HEI-2015 are shown in Table 1.

**Table 1:** Clinical characteristics at first visit of pregnant women included in the study (Rio de Janeiro, 2014-2017), and comparisons according to Healthy Eating Index-2015

Clinical characteristics	Whole group	Total HEI score		
		Low score (0.0-49.9 points)		p value
		YES	NO	
Age (years)	27.5 (18.0-35.0)	**26.8 (18.0-35.0)**	**28.0 (18-35)**	**0.012**
Gestational age (weeks)	9.0 (3.0-14.0)	9.0 (3.0-14)	9.0 (3.0-13.0)	0.860
First pregnancy	48%	47.8%	48.2%	0.507
BMI (kg/m^2^)	24.6 (15.0-49.1)	24.7 (15.6-49.1)	24.3 (15.6-40.1)	0.412
Underweight	11.3%	6.9%	4.5%	0.540
Normal weight	42.8%	49.0%	55.6%	
Overweight	28.7%	26.1%	26.3%	
Obesity	17.2%	12.2%	11.3%	
Alcohol drinker	1.0%	1.1%	0.7%	0.577

HEI: Healthy Eating Index-2015; BMI: body mass index.

The correlations between total scores and the scores for each HEI-2015 component with UIC, BMI and age are shown in Table 3. The UIC was slightly correlated with the components “total fruit”, “whole fruit” and “total vegetables”. Both age and BMI were also slightly correlated with “total fruits”, “whole fruits” and “total protein foods”, suggesting that higher BMI and younger age were correlated with lower scores. Age also tended to be positively correlated with the total HEI-2015 score.

**Table 3:** Adapted Healthy Eating Index-2015 – frequency of high and low scores for each component of HEI-2015, according to the respective urinary iodine concentration (UIC) subgroup and its association with IS in multivariate analysis, considering, in this case, the group with adequate UIC as the reference group

HEI-2015		Bivariate analysys*									Multivariate analysis**			
		Whole group (n = 418)	Insufficient UIC (<150 µg/L) (n = 101)	Adequate UIC (150-249 µg/L) (n = 146)	More than adequate UIC (250-499 µg/L) (n = 147)	Excessive UIC (≥500 µg/L) (n = 24)	p value***	Insufficient UIC (< 150 µg/L) (n = 101)	Adequate UIC (150-249 µg/L) (n=146)	More than adequate UIC (250-499 µg/L) (n = 147)	Excessive UIC (≥500 µg/L) (n = 24)
**Total score**														
	High score (90-100)	0	0	0				0	0	NE		RG		
	Low score (0-49.9)	275 (65.8%)	57 (56.4%)f	97 (66.4%)^a^				103 (70.1%)	18 (75.0%)	0.109	0.670 (0.39-1.15)	RG	1.17 (0.70-1.93)	1.48 (0.54-4.00)
**Adapted HEI-2015 components**														
**Total fruits**	High score (90-100)	138 (33%)	40 (39.6%)	49 (33.6%)				43 (29.3%)	2 (25.0%)	0.303	1.09 (0.63-1.89)	RG	0.82 (0.49-1.40)	0.66 (0.24-1.81)
	Low score (0-49.9)	212 (50.7%)	**41 (40.6%)** ^f^	**77 (52.7%)** ^a^				79 (53.7%)	**15 (62.5%)** ^e^	**0.098**	0.72 (0.42-1.23)	RG	1.05 (0.65-1.68)	1.48 (0.59-3.68)
**Whole fruits**														
	High score (90-100)	168 (40.2%)	**58 (57.4%)** ^a,f^	**50 (34.2%)** ^b^				54 (36.7%)	6 (25.0%)	0.001	2.24 (1.31-3.86)	RG	1.12 (0.68-1.84)	0.65 (0.24-1.80)
	Low score (0-49.9)	225 (53.8%)	**38 (37.6%)** ^f^	**83 (56.8%)** ^b,c^				**86 (58.5%)** ^g^	**18 (75.0%)** ^e^	**0.001**	**0.721 (0.424-1.227)**	**RG**	**1.05 (0.65-1.68)**	**1.48 (0.59-3.68)**
**Total vegetables**	High score (90-100)	41 (9.8%)	14 **(13.9%)**^f^	12 (8.2%)				15 (10.2%)	0 (0.0%)	0.177	1.78 (0.78-4.08)	RG	1.22 (0.54-2.75)	0.09 (0.00-∞)
	Low score (0-49.9)	335 (80.1%)	**75 (74.3%)**	**118 (80.8%)** ^c^				119 (81.0%)	23 (95.8%)	0.111	0.70 (0.78-1.30)	RG	1.03 (0.57-1.87)	5.3 (0.69-41.5)
**Greens**	High score (90-100)	357 (85.4%)	87 (86.1%)	128 (87.7%)				122 (83.0%)	20 (83.3%)	0.702	1.08 (0.50-2.35)	RG	0.65 (0.33-1.25)	0.63 (0.20-2.090
	Low score (0-49.9)	32 (7.7%)	5 (5.0%)	**8 (5.5%)** ^d^				18 (12.2%)	1 (4.2%)	0.078	0.64 (0.18-2.19)	RG	2.48 (1.03- 5.96)	0.78 (0.09-6.59)
**Whole grains**	High score (90-100)	20 (4.8%)	3 (3.0%)	9 (6.2%)			7 (4.8%)		1 (4.2%)	0.715	0.43 (0.11-1.64)	RG	0.79 (0.28-2.19)	0.69 (0.08-5.80)
	Low score (0-49.9)	382 (91.4%)	94 (93.1%)	132 (90.4%)			133 (90.5%)		23 (95.8%)	0.730	1.59 (0.61-4.15)	RG	1.07 (0.48-2.40)	2.38 (0.29-19.2)
**Dairy**	High score (90-100)	22 (5.3%)	4 (4.0%)	10 (6.8%)			6 (4.1%)		2.(8.3%)	0.584	0.4559 (0.17-1.87)	RG	0.59 (0.21-1.71)	1.31 (0.26-6.55)
	Low score (0-49.9)	342 (81.8%)	84 (83.2%)	118 (80.8%)			121 (82.3%)		19 (79.2%)	0.949	1.07 (0.56-1.91)	RG	1.04 (0.56-1.910	0.81 (0.28-2.40)
**Total protein foods**	High score (90-100)	177 (42.3%)	45 (44.6%)	57 (45.9%)			57 (38.8%)		8 (33.3%)	0.472	0.98 (0.58-1.65)	RG	0.79 (0.49-1.27)	0.59 (0.24-1.49)
	Low score (0-49.9)	137 (32.8%)	32 (31.7%)	**42 (28.8%)** ^d^			54 (36.7%)		9 (37.5%)	0.492	1.12 (0.70-1.96)	RG	1.35 (0.83-2.23)	1.47 (0.60-3.64)
**Seafood and plant proteins**	High score (90-100)	257 (61.5%)	62 (61.4%)	90 (61.6%)			91 (61.9%)		14 (58.3%)	0.990	0.99 (0.58-1.70)	RG	1.08 (0.67-1.74)	0.93 (0.38-2.24)
	Low score (0-49.9)	139 (33.3%)	33 (32.7%)	51 (34.9%)			46 (31.3%)		9 (37.5%)	0.885	0.89 (0.51-1.54)	RG	0.78 (0.48-1.29)	1.07 (0.43-2.62)
**Fatty acids**	High score (90-100)	56 (13.4%)	18 (17.6%)	14 (9.6%)^a^			20 (13.6%)		4 (16.7%)	0.289	0.68 (0.39-1.97)	RG	0.82 (0.49-1.37)	0.66 (0.27-1.64)
	Low score (0-49.9)	287 (68.7%)	67 (66.3%)	106 (72.6%)			99 (67.3%)		15 (62.5%)	0.604	0.68 (0.29-1.20)	RG	0.82 (0.49-1.37)	0.66 (0.27-1.64)
	**Moderation components**													
**Refined grains**	High score (90-100)	65 (15.6%)	17 (16.8%)	23 (15.8%)			20 (13.6%)		5 (20.8%)	0.787	1.13 (0.55-2.24)	RG	0.82 (0.42-1.60)	1.43 (0.48-4.28)
	Low score (0-49.9)	267 (63.9%)	58 (57.4%)^f^	92 (63.0%)			103 (70.1%)		14 (58.3%)	0.201	0.79 (0.47-1.35)	RG	1.4 (0.86-2.31)	0.79 (0.33-1.93)
**Sodium**	High score (90-100)	110 (26.3%)	33 (32.7%)	38 (26.0%)			35 (33.8%)		4 (16.7%)	0.291	0.13 (0.87-2.74)	RG	0.89 (0.51-1.52)	0.56 (0.20-1.77)
	Low score (0-49.9)	167 (40.0%)	39 (38.6%)	64 (43.8%)			54 (36.7%)		10 (41.7%)	0.647	0.77 (0.45-1.30)	RG	0.78 (0.48-1.25)	0.95 (0.40-2.31)
**Added sugars**	High score (90-100)	101 (24.2%)	26 (25.7%)	33 (22.6%)			35 (23.8%)		7 (29.6%)	0.880	1.16 (0.63-2.12)	RG	1.07 (0.62-1.85)	1.47 (0.56-3.88)
	Low score (0-49.9)	160 (38.3%)	35 (34.7%)	**66 (45.2%)** ^a,d^			50 (34.0%)		9 (37.5%)	0.198	0.66 (0.38-1.13)	RG	0.59 (0.36-0.95)	0.72 (0.29-1.75)
**Saturated fats**	High score (90-100)	153 (36.6%)	34 (33.7%)	56 (38.4%)			53 (36.1%)		10 (4.7%)	0.837	0.82 (0.48-1.41)	RG	0.87 (0.53-1.41)	1.13 (0.47-2.73)
	Low score (0-49.9)	195 (46.7%)	46 (45.5%)	73 (50.0%)			12 (50.0%)		10 (41.7%)	0.708	1.04 (0.59-1.81)	RG	0.83 (0.50-1.38)	1.26 (0.51-3.10)

In multivariate analysis results are shown in odds ratio (95% confidence intervals).

* Comparisons among all groups using the Chi-square test. Additionally, we used the adjusted Chi-square test for comparisons between groups, considering the p-value <0.017 as significant.

** Logistic binary regression was applied for multivariate analysis, considering the reference group as those with adequate UIC.

*** A p value < 0.05 was considered significant, and a p value of 0.05-0.10 was considered borderline in this context.

a: p borderline (0.0125 to 0.09) comparing adequate UIC and insufficient UIC;

b: p significant (<0.0125) comparing adequate and insufficient UIC;

c: p borderline (0.0125 to 0.09) comparing adequate and excessive UIC;

d: p borderline (0.0125 to 0.09) comparing the adequate with the subgroup “more than adequate” UIC;

e: p sig (<0.0125) comparing insufficient UIC and excessive UIC;

f: p borderline (0.0125 to 0.09) comparing insufficient UIC with “excessive” UIC.

g: p borderline (0.0125-0.09) comparing the group “more than adequate” with insufficient UIC. High score: 90-100 points; Low score: 0.0-49.9 points.

RG: reference group; NE: not estimated; UIC: urinary iodine concentration; NE: not evaluated; OD: odds ratio; CI: confidence interval.

Low scores (<50 points) were strongly present (>80%) when some components of HEI-2015, such as “total vegetables”, “whole grains”, and “dairy”, were considered, as demonstrated in Table 3.

Despite the absence of high pontuations in the total HEI-2015 in the whole studied group, regarding the specific components “greens” and “seafood and plant proteins”, those scores between 90.0 and 100 occurred in more than 50% of the cases (Table 3).

**Figure 3:**
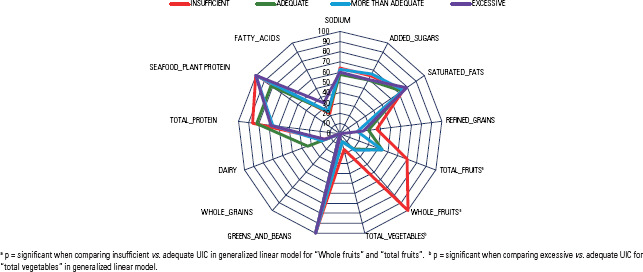
Graphic representation of the median scores of the HEI-2015 components according to the respective urinary iodine concentration of pregnant women.

The group with insufficient UICs had higher scores for “total fruits”, “total vegetables” and “whole fruits” than did those with adequate UICs, as demonstrated in Figure 3. With respect to “whole fruits” and “total fruits”, the group with insufficient iodine status had a higher frequency of high scores, whereas the group with excessive iodine status more frequently had worse scores (Table 2). A similar pattern, with a lower frequency of lower scores in the group with insufficient iodine status, was also detected in the refined grains (Table 3).

**Table 2:** Correlations between total scores, and the scores in each Healthy Eating Index-2015 component, with urinary iodine concentration, body mass index and age

	UIC (µg/L)		BMI (kg/m^2^)		Age (years)	
	r_s_	p value	r_s_	p value	r_s_	p value
HEI – Total score	-0.058	0.110	-0.047	0.174	**0.082**	**0.047**
**Total fruits**	**-0.128**	**<0.01**	**-0.120**	**<0.01**	**0.129**	**<0.01**
**Whole fruits**	**-0.136**	**<0.01**	**-0.152**	**<0.01**	**0.097**	**0.024**
**Total vegetables**	**-0.100**	**0.020**	0.050	0.175	0.138	<0.01
Greens	0.031	0.263	0.050	0.158	-0.120	<0.01
Whole grains	0.024	0.316	-0.080	0.050	-0.021	0.337
Dairy	0.030	0.268	0.000	0.499	0.018	0.360
**Total protein foods**	-0.018	0.357	**-0.090**	**0.040**	**0.088**	**0.05**
Seafood and plant proteins	0.012	0.403	-0.023	0.320	0.062	0.104
Fatty acids	0.063	0.070	0.04	0.186	-0.016	0.372
Refined grains	-0.017	0.017	-0.023	0.324	0.052	0.145
Sodium	-0.032	0.254	0.008	0.435	-0.048	0.164
Added sugar	0.01	0.440	-0.018	0.358	-0.003	0.478
Saturated fats	-0.013	0.265	-0.038	0.224	0.003	0.475

r_s_: Spearman’s correlation index; BMI: body mass index; UIC: urinary iodine concentration; HEI-2015: Healthy Eating Index-2015.

## DISCUSSION

The median HEI-2015 score in our population (45.8 points) was lower than that reported previously in different studies and populations ^(21^-^23)^. This may reflect the specific characteristics of the studied sample, which were derived from the National Health System (SUS), a program aimed at those subjects with socioeconomic vulnerability. Another point that should be mentioned is the high frequency (>60%) of women with the lowest scores for some components, such as “whole grains”, “total vegetables” and “dairy”, which reflects the poor alimentary habitus of the studied population. This finding may also reflect the socioeconomic conditions of the studied population, which were mainly composed of women who attended public basic health care units. We believe that it has an impact on diet quality since ultra-processed food, which is detrimental to fresh food, is less expensive and more assessable for this population.

The median UIC in the studied group was within the WHO’s recommended range for pregnant women (^12^), indicating a status of iodine sufficiency. However, a large variation in UIC, with a high prevalence of samples compatible with inadequate iodine status (either insufficient or excessive/more than sufficient), was detected. Elucidating the determinants of such variations would benefit clinical and socioeconomic strategies to minimize the impact of iodine insufficiency on pregnancy outcomes. Previously, we demonstrated, in the same population, that younger age, higher BMI and multiparity were associated with a greater risk for excessive or more than sufficient iodine (^3^). In the present study, despite this association being low, the diet quality analyses revealed that the scores of total fruit, whole fruit and total vegetables were inversely correlated with UIC. Additionally, the scores of total fruit, whole fruit and total protein foods were inversely correlated with BMI. However, the scores of total HEI, total fruit, whole fruit and total protein foods were directly correlated with age. These results are in accordance with our previous findings. It was speculated that those women probably had poorer diet quality and higher intake of salt or ultra-processed food. In addition, other authors reported that women with previous pregnancies were more likely to have poorer diet quality than those with first-time pregnancies (^24^). High iodine ingestion may be associated with an increased risk of subclinical hypothyroidism, which is likely related to autoimmune thyroid disease (^9^,^10^). In contrast, in a previous study with the same pregnant population group presented here, older women in their first pregnancy and with an adequate BMI should represent women with a higher risk for iodine insufficiency (^3^).

The findings of the present study support this hypothesis by showing that insufficient UIC was associated with better diet quality and higher HEI-2015 scores. As shown in Table 3, in the bivariate analysis, many parameters were related to iodine status in the high-score and low-score groups. When multivariate analysis was performed, “whole fruits” was independently related to iodine status in the high and low groups.

Notably, not only iodine insufficiency but also excess iodine may negatively impact thyroid function and pregnancy outcomes. In a previous study by our group (^11^), an increased risk for gestational diabetes (GDM) and hypertensive disorders of pregnancy (HDP) was also demonstrated when UIC was above the recommended range. We cannot affirm whether this association is related to diet quality, amount of salt intake or even thyroid dysfunction related to excessive iodine intake.

In this study, we could not demonstrate a direct association between the sodium component and UIC. However, we can assume that the lower scores for “whole fruits”, “total fruits” and “total vegetables” denote worse diet quality in pregnant women with excessive iodine status and are likely related to the high consumption of ultra-processed foods and, consequently, high intake of salt and sodium. Notably, greater consumption of ultra-processed foods, which may contain salt in their formulation (^18^), could constitute alternative sources of iodine. In contrast, better scores in “total fruits”, “whole fruits”, “refined grains” and “total protein” detected in pregnant women with iodine insufficiency may be related to better diet quality, which is commonly associated with a low intake of salt and ultra-processed food. The HEI-2015 is not an instrument for directly assessing the consumption of processed or ultra-processed food, but it is well known that the quality of a diet is inversely related to the consumption of these kinds of food ^(25^-^27]^.

Since 1982, the entire Brazilian population has received a minimum amount of iodine in table salt because of advances in public health policies. However, the WHO recommends that the maximum daily consumption of salt be less than five grams per person. However, according to the Brazilian Institute of Geography and Statistics (IBGE), the average salt consumption of Brazilians is 12 grams daily, a value that exceeds twice the recommended value ^(23^,^25^-^27)^. Because of this, a determination was published by the National Health Surveillance Agency (ANVISA) in the Official Gazette of the Union (DOU) of April 25, 2013, changing the iodation range of salt used in Brazil. According to the new rule, the addition of iodine to table salt ranges from 20-60 to 15-45 milligrams per kilo (mg/kg) of salt (^1^); the same is equivalent to 150-450 µg per 10 grams of salt. Importantly, a previous study by our group revealed that, in 98.5% of the table salts analyzed in this cohort, the iodine concentration was compatible with governmental recommendations (15-45 mg/kg) (^3^).

Studying other pathways to explain the impact of diet quality on thyroid function, not solely related to iodine consumption, is important. Limited consumption of fruit and vegetables is associated with an increased risk of “high total lipid peroxide levels in serum”, which could be related to autoimmune diseases, such as Hashimoto’s thyroiditis (HT) ^(24^-^27)^. Notably, our study has several limitations. First, as with all cross-sectional observational studies, causal inference is not possible. Diet quality may vary according to socioeconomic status, and as such, dietary patterns may vary substantially between high- and low-SES countries. Additionally, this study did not assess the amount of iodine intake from the ingested food. In Brazil, food composition tables do not include iodine content evaluations. Based on the impossibility of adequately assessing the amount of iodine intake according to the ingested food, this study focused on diet quality. The main objective of this study was to determine whether there is any relationship between diet quality and iodine status.

An important limitation of the present study is related to the fact that the results were gathered 7 years ago.However, supposing that the major source of iodine intake in our country is related to salt intake and that there has been no change in the governmental requirement for iodine fortification since that time, we believe that the data presented in the manuscript are still actual. Additionally, the study was conducted before the COVID-19 pandemic, which is a well-known factor that led to changes in nutritional behaviors ^(28^-^30)^. However, the major changes in nutritional behaviors during the COVID-19 pandemic were related to the lockdown (28-30), which occurred only in the earliest moments of the pandemic in Brazil. Importantly, this study did not evaluate the risk of iodine deficiency or excess, but rather the association between diet quality and urinary iodine concentration during a specific period was evaluated.

In conclusions these results suggest that diet quality is related to iodine status in the studied population. Pregnant women with better diet quality, especially those with high consumption of total protein foods, fruits and vegetables and low consumption of refined grains, may be at risk for iodine insufficiency. These groups likely benefit from iodine supplementation during pregnancy even when they live in a coastal state where table salt is fortified with iodine.

Furthermore, poor diet quality, related to the lower consumption of fruits and vegetables and high consumption of refined grains, was associated with excessive UIC during pregnancy, a condition that may lead to deleterious effects, especially if iodine supplementation is added to their prescription.

This study reinforces the importance of assessing the diet quality of pregnant women before conducting any kind of public health policy regarding supplementation during pregnancy. More prospective studies in different populations with different dietary exposures should be conducted to assess the reproducibility of these results in other regions.
