# Supplementary material for: Diet quality and its relationship with iodine status in pregnant women living in a Brazilian region where table salt is iodine-fortified according to public health policies
Source: Arch Endocrinol Metab. 2025 Jun 27;69(3):e240164. doi: 10.20945/2359-4292-2024-0164 (PMC12403729; doi:10.20945/2359-4292-2024-0164)
Supplement: SUPPLEMENTARY MATERIAL [file 2359-4292-aem-69-03-e240164-Supplementary_Material.pdf]

SUPPLEMENTARY MATERIAL

Supplementary Table 1. Clinical characteristics at first visit of pregnant women included in the study (Rio de Janeiro, 2014-2017), and comparisons according to their median urinary iodine concentration

Clinical characteristics	Whole group n = 199	Iodine status according to the median UIC of the pregnant women				p value*
		Insufficient (median UIC < 150 µg/L) n = 38	Adequate (median UIC 150-249 µg/L) n = 76	More than adequate (median UIC 250-499 µg/L) n = 80	Excessive (median UIC ≥500 µg/L) n = 5	
Age (years)	27.5 (18.0-35.0)	28.5 (18.0-35.0)	28 (18.0-35.0)	26 (18.0-35.0)	28 (19.0-33.0)	0.409
Gestational age (weeks)	9.0 (3.0-14.0)	9.0 (4.0-13.0)	9.0 (3.0-13.0)	9.0 (4.5-14.0)	8.0 (3.0-11.0)	0.612
First pregnancy	99 (48%)	22 (43%)	33 (46%)	37 (51.4%)	7 (58.3%)	0.410
BMI (kg/m^2)	24.6 (15.0-49.1)	25.3 (16.6-35.8)	24.4 (17.6-49.1)	25.2 (14.9-40.6)	26.4 (15.0-40.6)	0.857
Underweight	46 (11.3%)	5.6%	13.3%	12.8%	0.0%	0.688
Normal weight	174 (42.8%)	44.4%	42.7%	35.9%	40.0%	
Overweight	117 (28.7%)	36.1%	30.7%	29.5%	20.0%	
Obesity	70 (17.2%)	13.9%	13.3%	21.8%	40.0%	
Alcohol drinker	4 (1.0%)	0.0%	0%	3.8%	0.0%	0.199

BMI: body mass index; UIC: urinary iodine concentration.
Data are presented as the median (min-max) for continuous variables and as percentages for categorical variables.
* Kruskal-Wallis and Chi-square test for comparisons between all groups, respectively for continuous and categorical variables.

Copyright© AE&M all rights reserved.
